# Burden of early neonatal mortality in Sub-Saharan Africa. A systematic review and meta-analysis

**DOI:** 10.1371/journal.pone.0306297

**Published:** 2024-07-25

**Authors:** Natnael Moges, Anteneh Mengist Dessie, Denekew Tenaw Anley, Melkamu Aderajew Zemene, Natnael Atnafu Gebeyehu, Getachew Asmare Adella, Gizachew Ambaw Kassie, Misganaw Asmamaw Mengstie, Mohammed Abdu Seid, Endeshaw Chekol Abebe, Molalegn Mesele Gesese, Yenealem Solomon Kebede, Sefineh Fenta Feleke, Tadesse Asmamaw Dejenie, Natnael Amare Tesfa, Wubet Alebachew Bayih, Ermias Sisay Chanie, Berihun Bantie

**Affiliations:** 1 Department of Pediatrics and Child Health Nursing, College of Health sciences, Debre Tabor University, Debre Tabor, Ethiopia; 2 Department of Public Health, College of Health Sciences, Debre Tabor University, Debre Tabor, Ethiopia; 3 Department of Midwifery, College of Medicine and Health Science, Wolaita Sodo University, Wolaita Sodo, Ethiopia; 4 Department of Reproductive Health and Nutrition, School of Public Health, Woliata Sodo University, Woliata Sodo, Ethiopia; 5 Department of Epidemiology and Biostatistics, School of Public Health, Woliata Sodo University, Woliata Sodo, Ethiopia; 6 Department of Biochemistry, College of Health Sciences, Debre Tabor University, Debre Tabor, Ethiopia; 7 Unit of Physiology, Department of Biomedical Science, College of Health Science, Debre Tabor University, Debre Tabor, Ethiopia; 8 Department of Medical Laboratory Science, College of Health Sciences, Debre Tabor University, Debre Tabor, Ethiopia; 9 Department of Public Health, College of Health Sciences, Woldia University, Woldia, Ethiopia; 10 Department of Medical Biochemistry, College of Medicine and Health Sciences, University of Gondar, Gondar, Ethiopia; 11 School of Medicine, College of Health Science, Woldia University, Woldia, Ethiopia; 12 Department of Epidemiology and preventive Medicine, School of Public Health and Preventive Medicine, Faculty of Medicine, Nursing and Health Sciences, Monash University, Melbourne, Victoria, Australia; 13 Department of Comprehensive Nursing, College of Health Sciences, Debre Tabor University, Debre Tabor, Ethiopia; University of Ottawa, CANADA

## Abstract

**Background:**

Globally, with a neonatal mortality rate of 27/1000 live births, Sub-Saharan Africa has the highest rate in the world and is responsible for 43% of all infant fatalities. In the first week of life, almost three-fourths of neonatal deaths occur and about one million babies died on their first day of life. Previous studies lack conclusive evidence regarding the overall estimate of early neonatal mortality in Sub-Saharan Africa. Therefore, this review aimed to pool findings reported in the literature on magnitude of early neonatal mortality in Sub-Saharan Africa.

**Methods:**

This review’s output is the aggregate of magnitude of early neonatal mortality in sub-Saharan Africa. Up until June 8, 2023, we performed a comprehensive search of the databases PubMed/Medline, PubMed Central, Hinary, Google, Cochrane Library, African Journals Online, Web of Science, and Google Scholar. The studies were evaluated using the JBI appraisal check list. STATA 17 was employed for the analysis. Measures of study heterogeneity and publication bias were conducted using the I^2^ test and the Eggers and Beggs tests, respectively. The Der Simonian and Laird random-effect model was used to calculate the combined magnitude of early neonatal mortality. Besides, subgroup analysis, sensitivity analysis, and meta regression were carried out to identify the source of heterogeneity.

**Results:**

Fourteen studies were included from a total of 311 articles identified by the search with a total of 278,173 participants. The pooled magnitude of early neonatal mortality in sub-Saharan Africa was 80.3 (95% CI 66 to 94.6) per 1000 livebirths. Ethiopia had the highest pooled estimate of early neonatal mortality rate, at 20.1%, and Cameroon had the lowest rate, at 0.5%. Among the included studies, both the Cochrane Q test statistic (χ2 = 6432.46, P <0.001) and I^2^ test statistic (I^2^ = 99.80%, p <0.001) revealed statistically significant heterogeneity. Egger’s weighted regression (p <0.001) and funnel plot show evidence of publication bias in this meta-analysis.

**Conclusion:**

This review demonstrated that the pooled magnitude of early neonatal mortality in sub-Saharan Africa is substantial. Therefore, governmental and nongovernmental agencies, international organizations, healthcare providers and institutions and academic and research institutions should give a due attention and design strategies to reduce early neonatal mortality in Sub-Saharan Africa.

## Introduction

Early neonatal mortality(ENM) refers to the death of a newborn within the first 7 days of life [1]. In the first month of life, 2.4 million children worldwide perished in 2020. Approximately 6700 newborns die per day, which accounts for 47% of all child fatalities under the age of five, up from 40% in 1990 [2].

Attempts have been made all around the world and in individual countries to avoid the death of newborns. It was included in the ’unfinished agenda’ of the Millennium Development Goals (MDGs) and still is part of the Sustainable Development Goals (SDGs). Target two of the SDG three aims to stop preventable deaths of newborns by 2030, with all countries striving to lower neonatal mortality to 12 per 1000 live births [3]. Despite this, Sub-Saharan Africa(SSA) has the highest neonatal mortality rate 27/1000 live births in the world, accounting for 43 percent of all newborn deaths worldwide [2].

About three-fourths of newborn deaths happen during the first week of life, and in 2019, around one million babies died within the initial day of life. The main causes of these deaths were preterm birth, birth-related problems (not breathing at the time of birth), infections, and birth defects [2].

It seems that the rate of early neonatal mortality varies a lot from country to country and region to region. It looks like geographical location, economic development and healthcare infrastructure of the area all affect the early neonatal mortality rate (ENMR) [4]. Tanzania had an ENMR of 18 deaths per 1000 live births over the course of five years, and Ghana had a very early neonatal mortality rate of 9 out of every 1000 live births [5, 6].

It is vital to give details to important organizations about the burden of early neonatal mortality in Sub-Saharan Africa. This review can help the government, policy-makers, health professionals, researchers, communities and non-governmental organizations to reduce the burden of early neonatal mortality and to do more research. Therefore, Up-to-date evidence with pooled estimates is required to understand the burden of early neonatal mortality in SSA. Hence, this study aimed to pool findings reported on the magnitude of ENM in SSA to contribute reliable evidence that would inform newborn and infant health policy and practice.

## Materials and methods

### Reporting of the findings and review registration

Preferred Reporting Items for Systematic Reviews and Meta-Analyses(PRISMA) statements were used to report the current systematic review and meta-analysis [7] (S1 Table). The review protocol was registered in PROSPERO with the registration ID of CRD42023432975.

### Search strategies

We looked through numerous databases up until June 8, 2023, including PubMed/MEDLINE, PubMed Central, Hinary, Google, Cochrane Library, African Journals Online, Web of Science, and Google Scholar. These databases were selected as they index health and medical-related research. We even looked through the articles’ reference lists. The search keywords included free text keywords and Medical Subject Headings (**MeSH**) using Boolean operators, truncation, wildcards, and phrases in various databases. Using phrases from the Medical Subject Heading (MeSH), we conducted the primary search on PubMed. The same terms were searched for across all databases, and we used Google and Google Scholar to find any additional information we could (S2 Table).

### Eligibility criteria

Both published and unpublished articles of any time period or study design that report the magnitude of early neonatal mortality in Sub-Saharan African countries were included. Case studies, panel discussions, editorials, anonymous reports, and research that could not be accessed after two email communications to the principal author were excluded. In addition, studies were disregarded if they are published other than English Language.

### Outcome measurements

Early neonatal mortality is the term used to describe the death of a newborn within the initial seven days of life [8]. The major outcome of this review is to estimate the pooled magnitude of early neonatal mortality in SSA countries. Early neonatal mortality rate is computed by dividing number of early neonatal deaths (within seven days of birth) by the total number of live births [9].

### Quality assessment

The JBI quality appraisal checklist was used to evaluate the quality of each study [10]. The JBI critical appraisal checklist (which has nine items) was adapted for the studies reporting the prevalence data (S1 Checklist). Using the framework, two reviewers (NM, BB) independently evaluated the quality of each study. During the evaluation of quality, disagreements between reviewers were resolved by using the average score of the two reviewers. In the end, if the study received five or more points on all quality assessment items, it was deemed low risk [11].

### Study selection and data abstraction

In order to remove duplicate studies from the databases, we used the Endnote 21 program. After that, we used the titles and abstracts to select the studies that would be included. Two people (NM and BB) read through the full text studies and entered the material into a predetermined format to make sure we were receiving all the pertinent data. Any potential conflicts of interest were discussed in order to resolve them. If required, the lead investigator was also called. Data extraction took into account the first author, sample size, study country, cases, study methodology, publication year, prevalence period, and prevalence of early infant mortality. The prevalence data from all the studies had to be translated to per 1000 livebirths to ensure that everything was consistent. The review’s findings were then presented using per 1000 prevalence rates.

### Meta analysis

The data were taken out of Microsoft Excel and exported to STATA V.17 Statistical Software for further analysis. The statistical heterogeneity between studies was examined using the I^2^ statistic, and heterogeneity was visualized using a forest plot [12]. This demonstrated significant study heterogeneity (p< 0.001). Thus, a random-effect meta-analysis technique was used to ascertain the pooled prevalence of early neonatal mortality [13, 14]. Based on specific characteristics (study nation, study design), subgroup analysis was carried out. To determine the impact of a single study on the meta-total analysis’s estimate, a sensitivity analysis was conducted. To pinpoint the cause of heterogeneity, meta-regression analysis was taken into consideration.

### Assessment of publication bias

A funnel plot was used to depict the publication bias graphically. Statistics from the Egger’s regression test and the Begg’s test were utilized to formally identify publication bias [15, 16]. As a result, publication bias was defined as a p of< 0.05.

## Results

### Study selection

**Fig 1:**
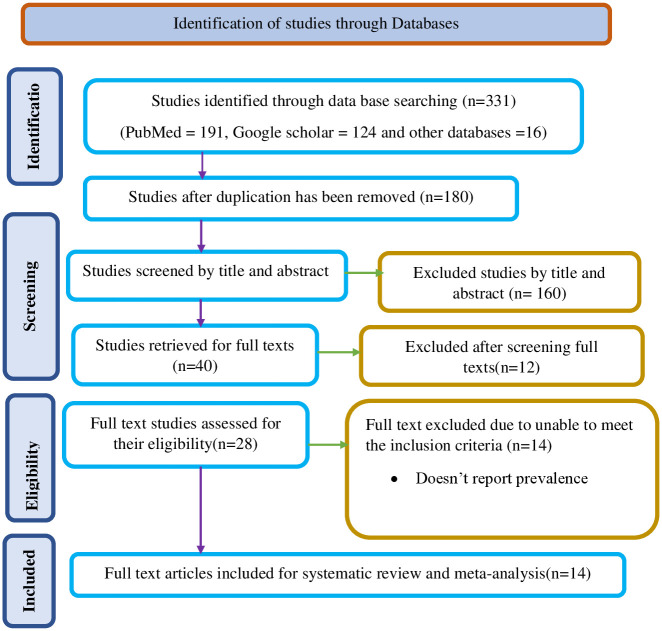
Study selection flow diagram; a figure adapted from the PRISMA group statement for this review. PRISMA, Preferred Reporting Items for Systematic Reviews and Meta Analyses.

Through searches on databases like PubMed, Google Scholar, and others, initial study on the extent of early newborn death turned up 311 papers. 131 of them were discarded because of duplicate articles. After careful consideration of the titles and abstracts of the remaining 180 studies, 140 was eliminated as being inappropriate for our inquiry. The remaining 40 studies’ whole texts were read in their entirety. The 14 studies that satisfied the inclusion criteria were included in this systematic review and meta-analysis [5, 8, 17–28] (Fig 1).

### Characteristics of original studies

The included studies were either cross-sectional (n = 8), retrospective (n = 4) or prospective studies (n = 2) [5, 8, 17–28]. Of all studies five studies were conducted in Ethiopia [8, 17, 25, 27, 28], two in Nigeria [19, 23], two in Ghana [5, 21], two in Tanzania [22, 26], one in Cameroon [18], one in Central Africa [20] and one in Malawi and Zambia [24]. All studies in this review were published in the year between 2009 and 2023. In addition, seven studies were hospital based and other seven articles were population based (Table 1).

**Table 1:** The characteristics of articles included in systematic review and meta-analysis 2023.

Firtst Author	Year	Country	Study design	Sample size	Cases	Prevalence/1000	prevalence period	Study setting	Study quality
Chelo et al [18]	2012	Cameroon	Cross -sectional	1623	8	5	2007–2008	Hospital based	Low risk
Engmann et al [20]	2009	Central Africa	Prospective	8230	263.4	32	2005–2007	Hospital based	Low risk
Tamir et al [27]	2023	Ethiopia	Cross -sectional	10525	440	42	--------	Population based	High risk
Ahmed et al [17]	2023	Ethiopia	Retrospective	765	99	129	2019–2021	Hospital based	Low risk
Tesfay et al [8]	2022	Ethiopia	Cross -sectional	3814	2190	574	---------	Population based	Low risk
Worku et al [28]	2012	Ethiopia	Retrospective	3789	881	233	2001–2005	Hospital based	Low risk
Mc Kinnon et al [25]	2014	Ethiopia	Cross -sectional	7668	231	30	----------	Population based	Low risk
Engmann et al [21]	2012	Ghana	Cross -sectional	20497	293	14	2002–208	Population based	Low risk
Avoka et al [5]	2018	Ghana	Retrospective	811	7.3	9	2013–2014	Hospital based	Low risk
Ezeh et al [23]	2017	Nigeria	Cross -sectional	63844	1749	27	---------	Population based	Low risk
Dahiru et al [19]	2015	Nigeria	Cross -sectional	119024	3772	32	---------	Population based	Low risk
Ersdal et al [22]	2012	Tanzania	Prospective	4720	49	10	2009	Hospital based	Low risk
Shayo et al [26]	2022	Tanzania	Retrospective	20250	369	18	2015–2019	Hospital based	Low risk
Lohela et al [24]	2012	Malawi&Zambia	Cross -sectional	12613	294	23	-----------	Population based	Low risk

### Quality of the studies

JBI quality appraisal guidelines were utilized to assess each included study’s effectiveness. The evaluation checklist for prevalence studies, which consists of nine questions and items with yes/no, ambiguous, or not applicable answers, was used to assess 14 papers. Based on the JBI descriptions for each item, the quality evaluation grade for all commodities was determined. The quality ratings of the studies ranged from four to nine as a consequence. No study had a discernible chance of being of low quality, with the exception of one that received four (S3 Table).

### Meta analysis

#### Magnitude of early neonatal mortality

**Fig 2:**
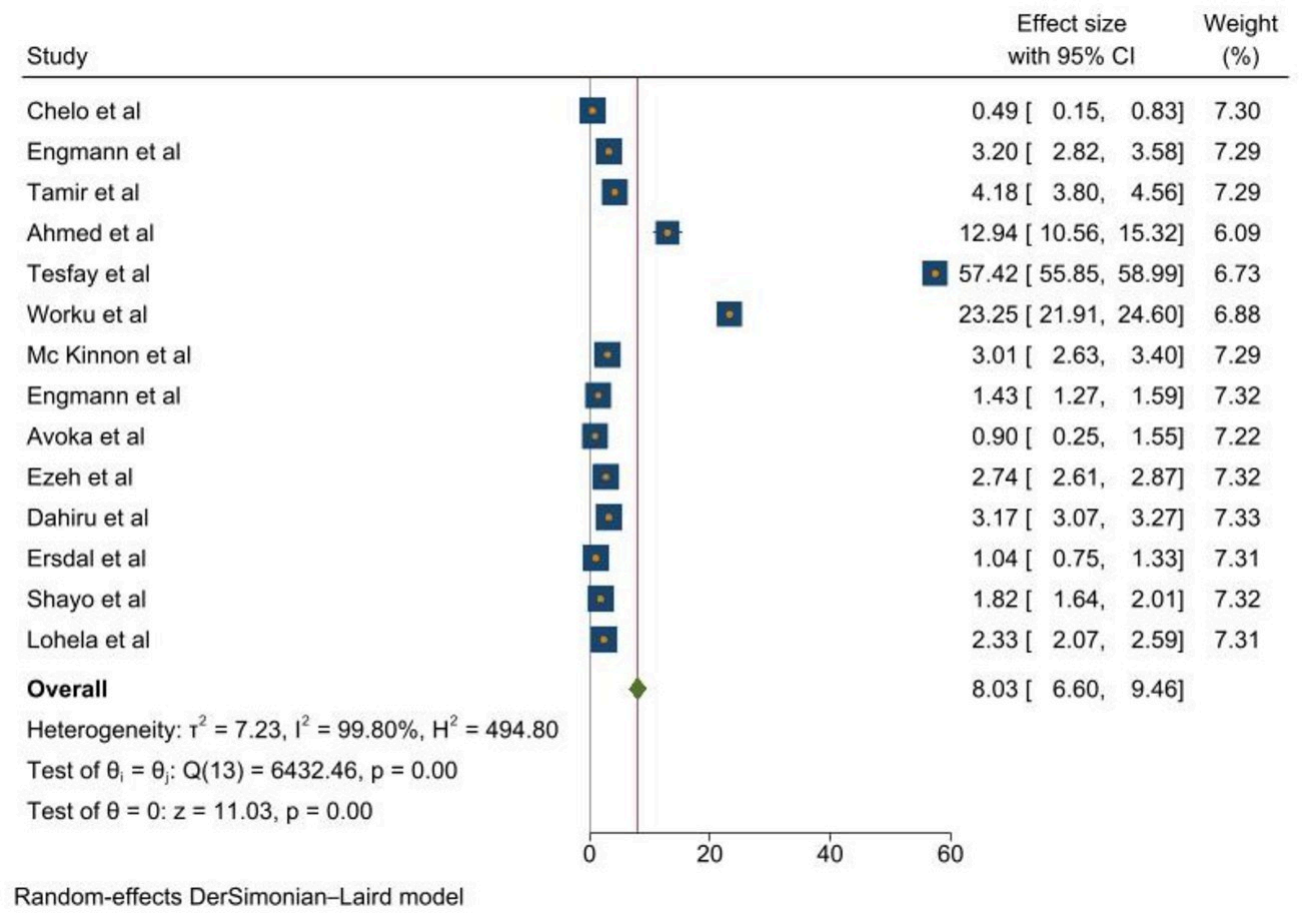
Forest plot showing the pooled prevalence of early neonatal mortality in sub-Saharan Africa,2023.

The pooled magnitude of early newborn mortality in the current meta-analysis was 80.3 per 1000 livebirths (95% CI 6.60% to 9.46%). The statistically significant heterogeneity between the studies was displayed via a forest plot. In order to combine the total prevalence of the studies, the random-effect meta-analysis approach was used (Fig 2).

### Sub group analysis

To determine how the prevalence varied among the studies, subgroup analysis depending on the study country and study methodology was conducted. To determine the combined magnitude of each country in Africa, a subgroup analysis depending on the study country was conducted. Ethiopia 20.1 (95% CI 9.14–31.15), Central Africa 3.2 (95% CI 2.82–3.58), and Nigeria 3 (95% CI 2.54–3.38) also had high pooled prevalences of early newborn death (Table 2). Statistically significant country-level heterogeneity was found in the current review (p<0.001, I2 = 99.80%). Because it is more conservative than the inverse variance method, the Der Simonian and Laird’s (D+L) pooled prevalence method was taken into consideration. There was a substantial difference across the nations (p <0.001).

**Table 2:** The pooled prevalence of early neonatal mortality among sub-Saharan African countries.

Country	Prevalence in % (95% CI)
Cameroon	0.5(0.15–0.83)
Central Africa	3.2(2.82–3.58)
Ethiopia	20.1(9.14–31.15)
Ghana	1.3(0.78–1.75)
Malawi& Zambia	2.3(2.07–2.59)
Nigeria	3(2.54–3.38)
Tanzania	1.4(0.67–2.21)
D+L pooled estimate	8.03(6.60–9.46)

D+L, Der Simonian and Laird; ES, Effect Size

**Fig 3:**
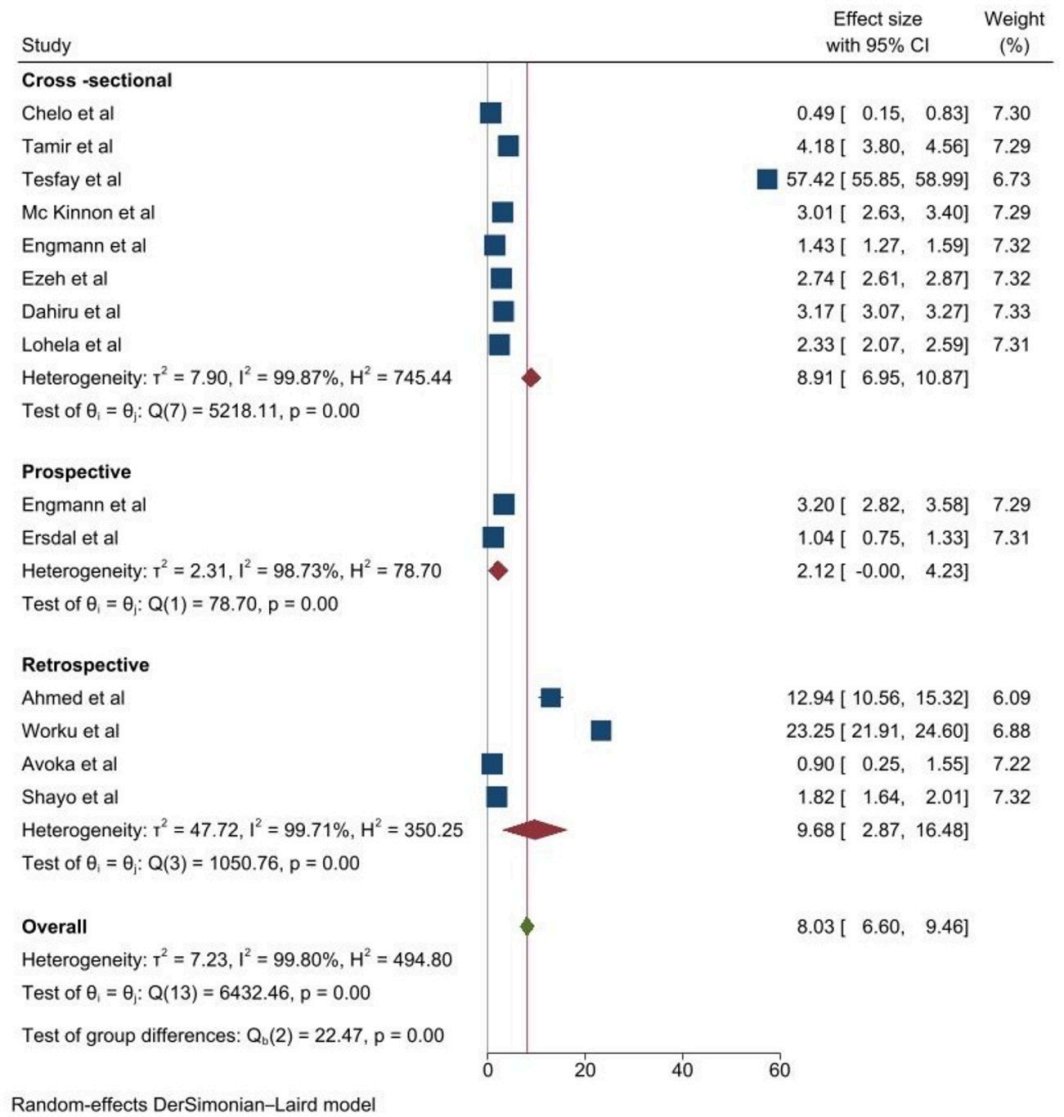
Subgroup analysis based on study design in sub-Saharan Africa. ES, Effect Size.

Subgroup analysis based on study design, using the D+L method (p<0.001, I^2^ = 99.80%) the magnitude of early neonatal mortality for cross-sectional studies was 8.91% and for prospective studies was 9.68% (Fig 3).

### Meta regression analysis

The sample size (p<0.001), year of publication (p<0.001), study country (p<0.001), study design (p = 0.868), and number of cases (p<0.001) were examined for the cause of heterogeneity in this systematic review and meta-analysis. For the source of heterogeneity, sample size, year of publication, number of cases, and research nation were important factors.

### Sensitivity analysis

**Fig 4:**
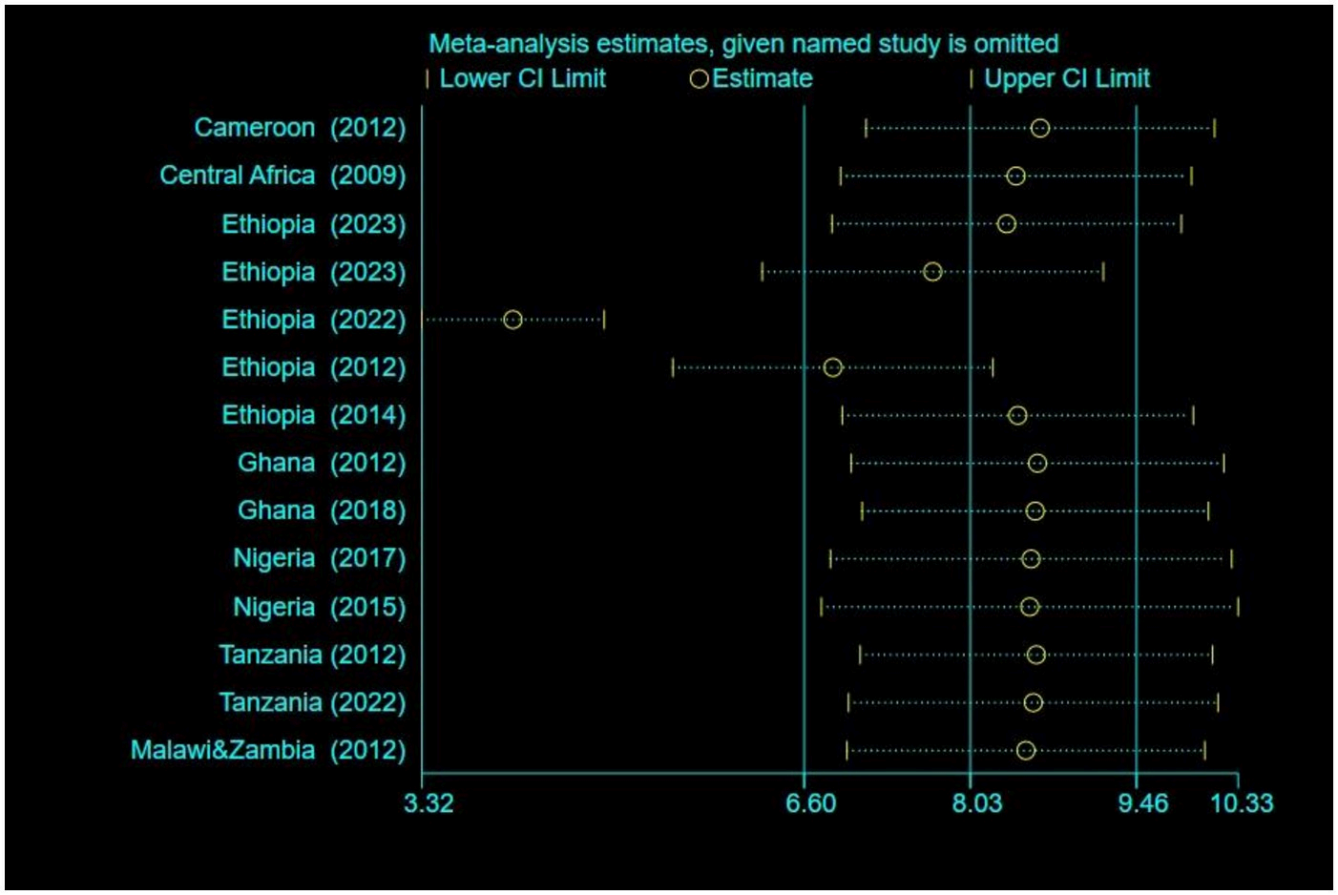
A sensitivity analysis for the pooled prevalence using the metaninf commands of STATA, showed no single study had a significant effect on the pooled estimate.

We did a leave-one-out sensitivity analysis with metaninf command to figure out how big of an effect one research had on the overall outcome. We observed that the conclusions weren’t greatly impacted by one study, since the point estimate of the omitted analysis stayed within the CI of the combined analysis, and the overall heterogeneity wasn’t dramatically changed. With a pooled estimate of 8.03% (95% CI 6.60% to 9.46%), this produced findings that were comparable to those we had previously discovered (Fig 4).

### Publication bias

**Fig 5:**
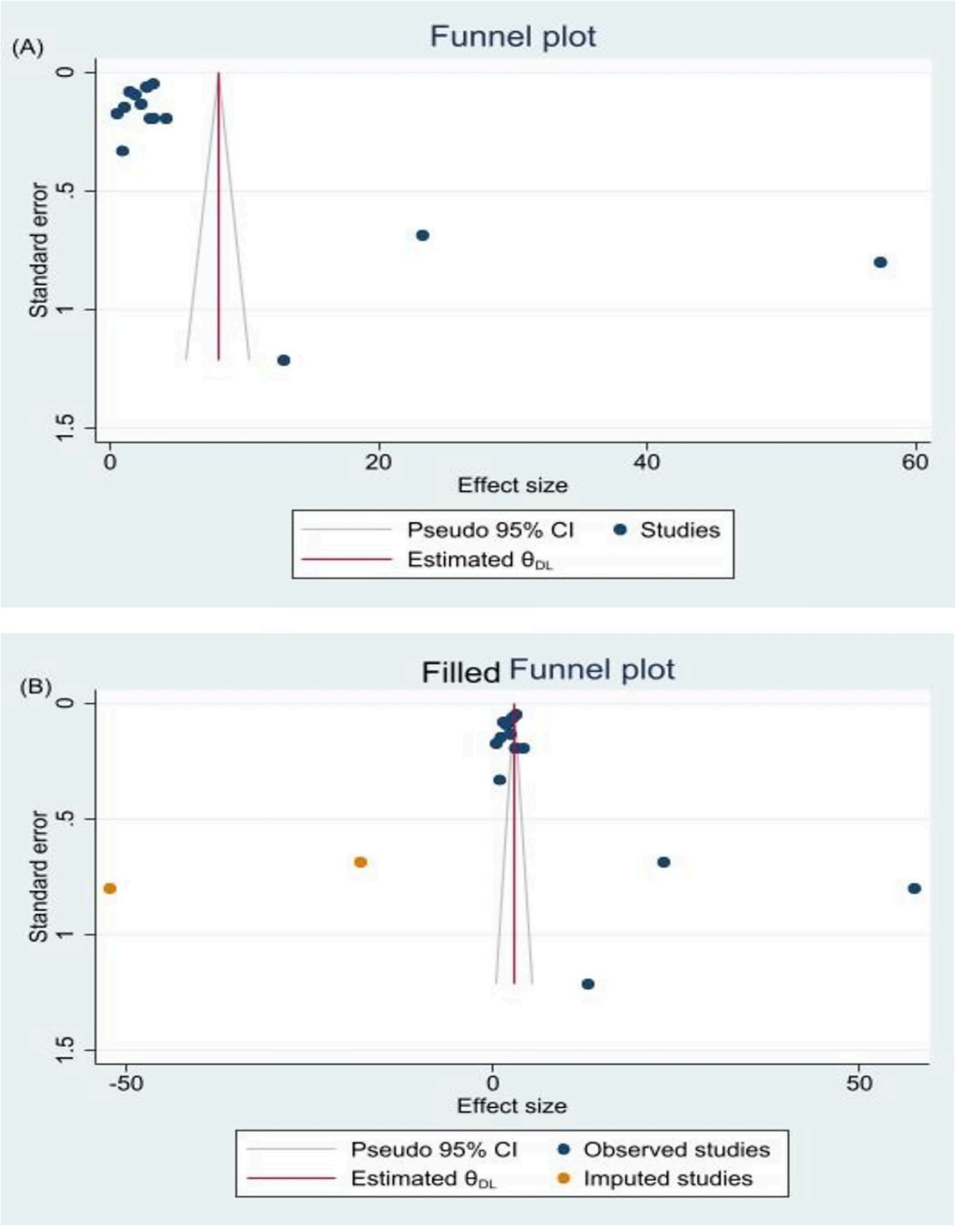
(A) A funnel plot with a pseudo 95% confidence limit used to test for publication bias. (B) A funnel plot with a pseudo 95% confidence limit after a trim-and-fill analysis in which two studies have been imputed.

Using Egger’s regression tests, publication bias was examined (B-coefficient of bias: 28.9; P = 0.0034). Uneven distribution could be seen in the funnel plot (Fig 5A and 5B). Additionally, the Egger and Begg tests clearly demonstrated publication bias (p< 0.05). A trim and fill study was then conducted. We used the run L0 estimator to fill in two trials, and the trim and fill analysis gave a pooled prevalence of 2.92 (95% CI: 1.11–4.73) with the two studies added.

## Discussion

Early neonatal mortality (ENM) refers to the death of a newborn within the first 7 days of life. It is acritical period in an infant’s life and the risk of mortality is high during this period. The aim of this systematic review and meta-analysis is to estimate the pooled magnitude of early neonatal mortality in sub-Saharan Africa.

Owing to the paucity of data pertaining to early neonatal mortalities, an assessment was conducted drawing from studies conducted across eight sub-Saharan African countries. Previous investigations have endeavored to elucidate the prevalence of early neonatal mortality in diverse countries, albeit yielding disparate findings, thereby lacking conclusive evidence regarding the overall incidence in Africa. This comprehensive review and analysis elucidate the widespread occurrence of early neonatal mortality across African nations, furnishing crucial insights for policymakers, clinicians, and stakeholders concerned with addressing this pressing public health concern.

The present review revealed that the pooled magnitude of early neonatal mortality is 80.3 per 1000 live births with the range of 66%-94.6%. This result implies that the burden of early neonatal mortality in sub-Saharan Africa is substantial. This could be related to causes of neonatal mortality in Africa are multifactorial and include poor quality of healthcare governance, inadequate health financing and human health resources, and poor socioeconomic status [29].

Moreover, this study showed that there’s a huge difference in the rate of early neonatal mortality between sub-Saharan Africa countries. We did a subgroup analysis by country and design. Ethiopia had the highest rate, at 20.1%, and Cameroon had the lowest rate. Nigeria had 3%, Ghana 1.3%, central Africa 3.2%, Tanzania 1.4%, Malawi and Zambia 2.3%. The difference might be some countries may have a good tracking system for births, but others might not. This could lead to an underestimation of the amount of early neonatal mortality in sub-Saharan Africa. Variations in access to antenatal screening and terminations (refers to ending pregnancy for medical reasons) also affected the estimates of the prevalence. For example, if there are a lot of terminated cases in one country, the prevalence at birth would likely be lower. Studies have shown that there can be big differences in prevalence estimates of early neonatal mortality between countries [30]. In Cameroon, the lowest prevalence might be due to the detection methods, reporting and recording system, and the number of terminated cases. Additionally, cultural factors including traditional values and practices can affect the health of newborns [31].

This review will be useful in helping African nations strengthen their prevention and control efforts to reduce neonatal mortality. To decide which interventions to prioritize, they might need to adjust their clinical and policy guidelines since some countries have different estimates of early neonatal mortality. It would be great if African nations could pass laws to make sure their food is fortified with folic acid, plus they should set up or upgrade their surveillance systems to keep an eye on pregnancy-related outcomes. This study clearly demonstrates how critical and common the burden of early neonatal mortality is in SSA, which should help decision-makers, health workers, and everyone else who’s downplayed this issue understand the seriousness of the problem.

Distinguishing the heavy burden in SSA should lead policymakers to create effective control and prevention strategies, with the ultimate goal of reducing early neonatal mortality and conducting further research. The differences in prevalence estimates among countries could inform clinical and policy guidelines for prioritizing interventions and maintaining surveillance systems that track or monitor all pregnancy outcomes and all births in SSA. Moreover, information gained from this review could help design and develop future research. It would also assist additional clinical studies in focusing on the risk factors, prevention and interventions. Ultimately, this review should help prevention and control programs decrease the burden of the early neonatal mortalities across Sub Saharan African countries.

This review has the following limitations.

For example, the estimates may not be accurate since abortions (termination of pregnancy <28 week of gestation in many developing nations) weren’t included. Plus, the variance in the sample sizes of the studies may have an effect on the pooled estimates. Also, the diversity between countries may make SSA burden seem smaller than it actually is. In addition, studies published in a language other than English and non-peer-reviewed articles may have been missed as only peer-reviewed articles published in English were included. Moreover, the expected report may be impacted by the diversity in study designs, sample sizes, study locations, and publication years. Thus, the interpretation of these study findings should take into account this variabilityIn conclusion, it was found that early newborn mortality is significantly higher in sub-Saharan Africa. Ethiopia, Nigeria, and central Africa were shown to have high rates of early infant mortality. We also want to inform decision-makers so they can put in place effective preventative and control measures. Besides, more primary and in-depth research is needed to better understand the causes and the true scope of early neonatal mortality. and support preventive actions for causes that can be avoided in sub-Saharan Africa.

## Supporting information

S1 Dataset (XLSX)

S1 Table PRISMA 2020 checklist. (DOCX)

S2 Table PubMed searching methods. (DOCX)

S3 Table Quality assessment of studies. (DOCX)

S1 Checklist JBI critical appraisal checklist. (DOCX)
