# Supplementary material for: Burden of early neonatal mortality in Sub-Saharan Africa. A systematic review and meta-analysis
Source: PLoS One. 2024 Jul 25;19(7):e0306297. doi: 10.1371/journal.pone.0306297 (PMC11271883; doi:10.1371/journal.pone.0306297)
Supplement: S2 Table — (DOCX) [file pone.0306297.s003.docx]

| **S.no.** | **Searching terms** | **Number of articles** |
| --- | --- | --- |
| **#1.** | **("Prevalence"[Mesh] OR Prevalence [tw] OR magnitude[tw])** | **483346** |
| **#2.** | **"Infant Mortality"[Mesh] OR “Early neonatal mortality” [tw] OR “early neonatal death” [tw]** | **7265** |
| **#3.** | **"Africa"[Mesh]** | **122859** |
|  | **#1 AND #2 AND #3** | **191** |

**Supplementary file 2: PubMed Searching Methods**
