# Supplementary material for: Burden of early neonatal mortality in Sub-Saharan Africa. A systematic review and meta-analysis
Source: PLoS One. 2024 Jul 25;19(7):e0306297. doi: 10.1371/journal.pone.0306297 (PMC11271883; doi:10.1371/journal.pone.0306297)
Supplement: S3 Table — (DOCX) [file pone.0306297.s004.docx]

Supplementary file 4: The quality status of studies based on JBI critical appraisal checklist for studies reporting prevalence data

| Studies | Appropri  ate sampling frame? | Appro  priate sampli ng? | Adequate sample size? | Detail setting descrip tion? | Analysis with sufficient coverage? | Valid method to identify the condition? | Reliable measure ment? | Appropri  ate statistical analysis? | Adequate response rate? | Total, out of 9 |
| --- | --- | --- | --- | --- | --- | --- | --- | --- | --- | --- |
| Chelo et al | Yes | N/A | yes | yes | Yes | Yes | UC | Yes | N/A | 8 |
| Engmann et al | Yes | Yes | Yes | Yes | Yes | UC | UC | Yes | UC | 6 |
| Tamir et al | Yes | UC | Yes | Yes | UC | UC | UC | Yes | UC | 4 |
| Ahmed et al | Yes | N/A | Yes | Yes | Yes | Yes | UC | Yes | N/A | 8 |
| Tesfay et al | Yes | N/A | Yes | Yes | Yes | UC | UC | Yes | N/A | 7 |
| Worku et al | Yes | N/A | Yes | Yes | Yes | Yes | UC | Yes | N/A | 8 |
| McKinnon et al | Yes | N/A | Yes | Yes | Yes | Yes | Uc | Yes | N/A | 8 |
| Engmann et al | Yes | N/A | Yes | Yes | Yes | Yes | Yes | Yes | N/A | 9 |
| Avoka et al | Yes | N/A | Yes | Yes | Yes | Yes | UC | Yes | N/A | 8 |
| Lohela et al | Yes | N/A | Yes | No | Yes | Yes | No | Yes | N/A | 7 |
| Ezeh et al | Yes | N/A | Yes | No | Yes | UC | No | Yes | N/A | 6 |
| Dahiru et al | Yes | Yes | Yes | Yes | Yes | Yes | UC | Yes | UC | 7 |
| Ersdal et al | Yes | N/A | Yes | Yes | Yes | Yes | UC | Yes | N/A | 8 |
| Shayo et al | Yes | N/A | Yes | Yes | No | UC | Yes | Yes | N/A | 7 |
