# Supplementary material for: Burden of early neonatal mortality in Sub-Saharan Africa. A systematic review and meta-analysis
Source: PLoS One. 2024 Jul 25;19(7):e0306297. doi: 10.1371/journal.pone.0306297 (PMC11271883; doi:10.1371/journal.pone.0306297)
Supplement: S1 Checklist — (DOCX) [file pone.0306297.s005.docx]

**Supplementary file 3: JBI Critical Appraisal Checklist**

**JBI Critical Appraisal Checklist for Studies Reporting Prevalence Data**

Reviewer ______________________ Date ____________________

Author ________________________ Year ___________ Record Number ______

**Yes No Unclear Not applicable**

1. Was the sample frame appropriate to address the  **□ □ □ □**

target population?

1. Were study participants sampled in an appropriate way?  **□ □ □ □**
2. Was the sample size adequate? **□ □ □ □**
3. Were the study subjects and the setting described in **□ □ □ □**

detail?

1. Was the data analysis conducted with sufficient coverage **□ □ □ □**

of the identified sample?

1. Were valid methods used for the identification of the **□ □ □ □**

condition?

1. Was the condition measured in a standard, reliable way **□ □ □ □**

for all participants?

1. Was there appropriate statistical analysis? **□ □ □ □**
2. Was the response rate adequate, and if not, was the low **□ □ □ □**

response rate managed appropriately?

**Overall appraisal: Include □ Exclude □ Seek further info □**

Comments (Including reason for exclusion)

__________________________________________________________________________________________________________________________________________________________________________

**JBI Critical Appraisal Checklist for Analytical Cross Sectional Studies**

Reviewer ______________________ Date ____________________

Author ________________________ Year ___________ Record Number ______

**Yes No Unclear Not applicable**

1. Were the criteria for inclusion in the sample clearly  **□ □ □ □**

defined?

1. Were the study subjects and the setting described in **□ □ □ □**

detail?

1. Was the exposure measured in a valid and reliable **□ □ □ □**

way?

1. Were objective, standard criteria used for **□ □ □ □**

measurement of the condition?

1. Were confounding factors identified? **□ □ □ □**
2. Were strategies to deal with confounding factors **□ □ □ □**

stated?

1. Were the outcomes measured in a valid and reliable **□ □ □ □**

way?

1. Was appropriate statistical analysis used? **□ □ □ □**

**Overall appraisal: Include □ Exclude □ Seek further info □**

Comments (Including reason for exclusion)

__________________________________________________________________________________________________________________________________________________________________________

**JBI Critical Appraisal Checklist for Case Control Studies**

Reviewer ______________________ Date ____________________

Author ________________________ Year ___________ Record Number ______

**Yes No Unclear Not applicable**

1. Were the groups comparable other than the

presence of disease in cases or the absence of  **□ □ □ □**

disease in controls?

1. Were cases and controls matched **□ □ □ □**

appropriately?

1. Were the same criteria used for identification of **□ □ □ □**

cases and controls?

1. Was exposure measured in a standard, valid and **□ □ □ □**

reliable way?

1. Was exposure measured in the same way for **□ □ □ □**

cases and controls?

of the identified sample?

1. Were confounding factors identified? **□ □ □ □**
2. Were strategies to deal with confounding factors **□ □ □ □**

stated?

1. Were outcomes assessed in a standard, valid and **□ □ □ □**

reliable way for cases and controls?

1. Was the exposure period of interest long enough **□ □ □ □**

to be meaningful?

1. Was appropriate statistical analysis used? **□ □ □ □**

**Overall appraisal: Include □ Exclude □ Seek further info □**

Comments (Including reason for exclusion)

__________________________________________________________________________________________________________________________________________________________________________

**JBI Critical Appraisal Checklist for Cohort Studies**

Reviewer ______________________ Date ____________________

Author ________________________ Year ___________ Record Number ______

**Yes No Unclear Not applicable**

1. Were the two groups similar and recruited from the **□ □ □ □** same population?
2. Were the exposures measured similarly to assign people **□ □ □ □**

to both exposed and unexposed groups?

1. Was the exposure measured in a valid and reliable way? **□ □ □ □**
2. Were confounding factors identified? **□ □ □ □**
3. Were strategies to deal with confounding factors **□ □ □ □**

stated?

1. Were the groups/participants free of the outcome at **□ □ □ □**

the start of the study (or at the moment of exposure)?

1. Were the outcomes measured in a valid and reliable **□ □ □ □**

way?

1. Was the follow up time reported and sufficient to be **□ □ □ □**

long enough for outcomes to occur?

1. Was follow up complete, and if not, were the reasons to **□ □ □ □**

loss to follow up described and explored?

1. Were strategies to address incomplete follow up **□ □ □ □**

utilized?

1. Was appropriate statistical analysis used? **□ □ □ □**

**Overall appraisal: Include □ Exclude □ Seek further info □**

Comments (Including reason for exclusion)

__________________________________________________________________________________________________________________________________________________________________________
